# Human Nasal Organoids Model SARS-CoV-2 Upper Respiratory Infection and Recapitulate the Differential Infectivity of Emerging Variants

**DOI:** 10.1128/mbio.01944-22

**Published:** 2022-08-08

**Authors:** Man Chun Chiu, Cun Li, Xiaojuan Liu, Wenjun Song, Zhixin Wan, Yifei Yu, Jingjing Huang, Ding Xiao, Hin Chu, Jian-Piao Cai, Kelvin Kai-Wang To, Kwok Yung Yuen, Jie Zhou

**Affiliations:** a Department of Microbiology, School of Clinical Medicine, Li Ka Shing Faculty of Medicine, The University of Hong Kong, Pokfulam, Hong Kong, China; b State Key Laboratory of Respiratory Disease, The First Affiliated Hospital of Guangzhou Medical University, Guangzhou, China; c State Key Laboratory of Emerging Infectious Diseases, The University of Hong Kong, Hong Kong, China; d Centre for Virology, Vaccinology and Therapeutics, Hong Kong Science and Technology Park, Hong Kong, China; e Carol Yu Centre for Infection, The University of Hong Kong, Pokfulam, Hong Kong, China; Johns Hopkins Bloomberg School of Public Health

**Keywords:** nasal organoids, airway organoids, SARS-CoV-2, ciliary damage, tight junction

## Abstract

The human upper respiratory tract, specifically the nasopharyngeal epithelium, is the entry portal and primary infection site of respiratory viruses. Productive infection of SARS-CoV-2 in the nasal epithelium constitutes the cellular basis of viral pathogenesis and transmissibility. Yet a robust and well-characterized *in vitro* model of the nasal epithelium remained elusive. Here we report an organoid culture system of the nasal epithelium. We derived nasal organoids from easily accessible nasal epithelial cells with a perfect establishment rate. The derived nasal organoids were consecutively passaged for over 6 months. We then established differentiation protocols to generate 3-dimensional differentiated nasal organoids and organoid monolayers of 2-dimensional format that faithfully simulate the nasal epithelium. Moreover, when differentiated under a slightly acidic pH, the nasal organoid monolayers represented the optimal correlate of the native nasal epithelium for modeling the high infectivity of SARS-CoV-2, superior to all existing organoid models. Notably, the differentiated nasal organoid monolayers accurately recapitulated higher infectivity and replicative fitness of the Omicron variant than the prior variants. SARS-CoV-2, especially the more transmissible Delta and Omicron variants, destroyed ciliated cells and disassembled tight junctions, thereby facilitating virus spread and transmission. In conclusion, we establish a robust organoid culture system of the human nasal epithelium for modeling upper respiratory infections and provide a physiologically-relevant model for assessing the infectivity of SARS-CoV-2 emerging variants.

## INTRODUCTION

The COVID-19 pandemic has caused unprecedented health and socioeconomic crisis since late 2019 (1, 2). Although COVID-19 patients have a risk of developing life-threatening pneumonia, most patients have mild to moderate symptoms, including cough, sore throat, and occasionally anosmia, due to the involvement of the upper respiratory tract. Clinically, pre-symptomatic and symptomatic COVID-19 patients have a high viral load in nasal swabs, indicating robust viral growth in the nasal epithelium and the high transmissibility of SARS-CoV-2 (3). The human upper respiratory tract, specifically the nasopharyngeal epithelium, is the entry portal and primary site for SARS-CoV-2 replication and transmission (4, 5). Particularly, nasal ciliated cells are the primary target for SARS-CoV-2 replication, especially in the early stage of infection (5).

The human airways, from the nasal cavity to terminal bronchioles, are covered with the airway epithelium, also known as the pseudostratified ciliated epithelium which consists of four major cell types: ciliated, goblet, club, and basal cells. Single-cell RNA sequencing technology has enabled an in-depth illumination of the global molecular profile of *in vivo* human cells, and remarkably advanced the prior understanding of human cells based on traditional characterization approaches. Single-cell sequencing studies of human airways demonstrated that the airway epithelium lining the nasal cavity differs from that covering the tracheobronchial region (6, 7). Goblet cells were more abundant in the nasal epithelium than in the tracheobronchial epithelium. In addition, the same cell types in the nose showed a differential gene expression profile from those in tracheobronchial airways, indicating region-specific sub-clusters of airway epithelial cell types in nasal and tracheobronchial compartments. Overall, nasal epithelial cells and the tracheobronchial counterparts have subtle but varied molecular profiles, which may lead to phenotypic variations accordingly.

Since late 2020, SARS-CoV-2 has undergone a series of mutations, including “variants of concern”(VOC) that affect virus transmissibility and antigenicity. Epidemiological data demonstrated that the Delta variant (B1.617.2), first identified in December 2020, was 55% more transmissible than the earlier Alpha variant (B1.1.7) (8). Subsequently, the Omicron variant (B1.1.529) emerged in November 2021; it rapidly swept across the world and has become the predominant variant (9). As the pandemic evolves, new variants would emerge inevitably. However, commonly used cell lines for studying human respiratory viruses such as A549 or Calu3 are implausible to simulate the multicellular complexity and functional diversity of human respiratory epithelium, let alone model respiratory infections. Air-liquid interface cultures of primary human airway and nasal epithelial cells develop mucociliary differentiation into pseudostratified airway epithelium. These epithelial cells have been used to study SARS-CoV-2 respiratory infection (2, 10–16). However, primary epithelial cells are unable to be long-term expanded due to limited *in vitro* proliferation capacity, the so-called “Hayflick limit” (17), which substantially restricts their application for routine research purposes.

We established the first adult stem cell-derived human lung organoids directly from primary lung tissues (18–22). These lung organoids were stably expanded for over 1 year, without any feeder cells and tedious cell purification procedures. We then induced proximal and distal differentiation in the long-term expandable lung organoids and generated mature airway and alveolar organoids that morphologically and functionally phenocopy the native airway and alveolar epithelium, respectively (18, 22). These physiologically active respiratory organoids have become a robust and popular research tool for studying SARS-CoV-2 and other respiratory viruses (22–26). As aforementioned, the airway epithelium in the nasal mucosa differs from that covering the tracheobronchial region (6, 7). Prior studies in human intestinal organoids, the first adult stem cell-derived organoids (27, 28), indicated that organoids generated from different intestinal segments retain the structural and functional characteristics from which they are derived (29). In this connection, we inferred that the organoids derived from nasal cells, if attainable, could simulate the nasal epithelium better than the airway organoids derived from lung tissues. In addition, the derivation of lung organoids relies on surgeons and physicians to obtain resected human tissues or bronchoalveolar lavage via invasive approaches. We attempted to procure nasal epithelial cells via a non-invasive and readily conductable procedure to generate nasal organoids. Mounting evidence indicates that the human upper respiratory tract, and particularly the nasal epithelium, are the primary site of SARS-CoV-2 replication and transmission (4, 5, 30). We hypothesize that the derived nasal organoids could provide an *in vitro* correlate of the native nasal epithelium for modeling SARS-CoV-2 upper respiratory infection and testing infectivity of emerging variants.

## RESULTS

### Derivation, differentiation, and characterization of nasal organoids.

**FIG 1:**
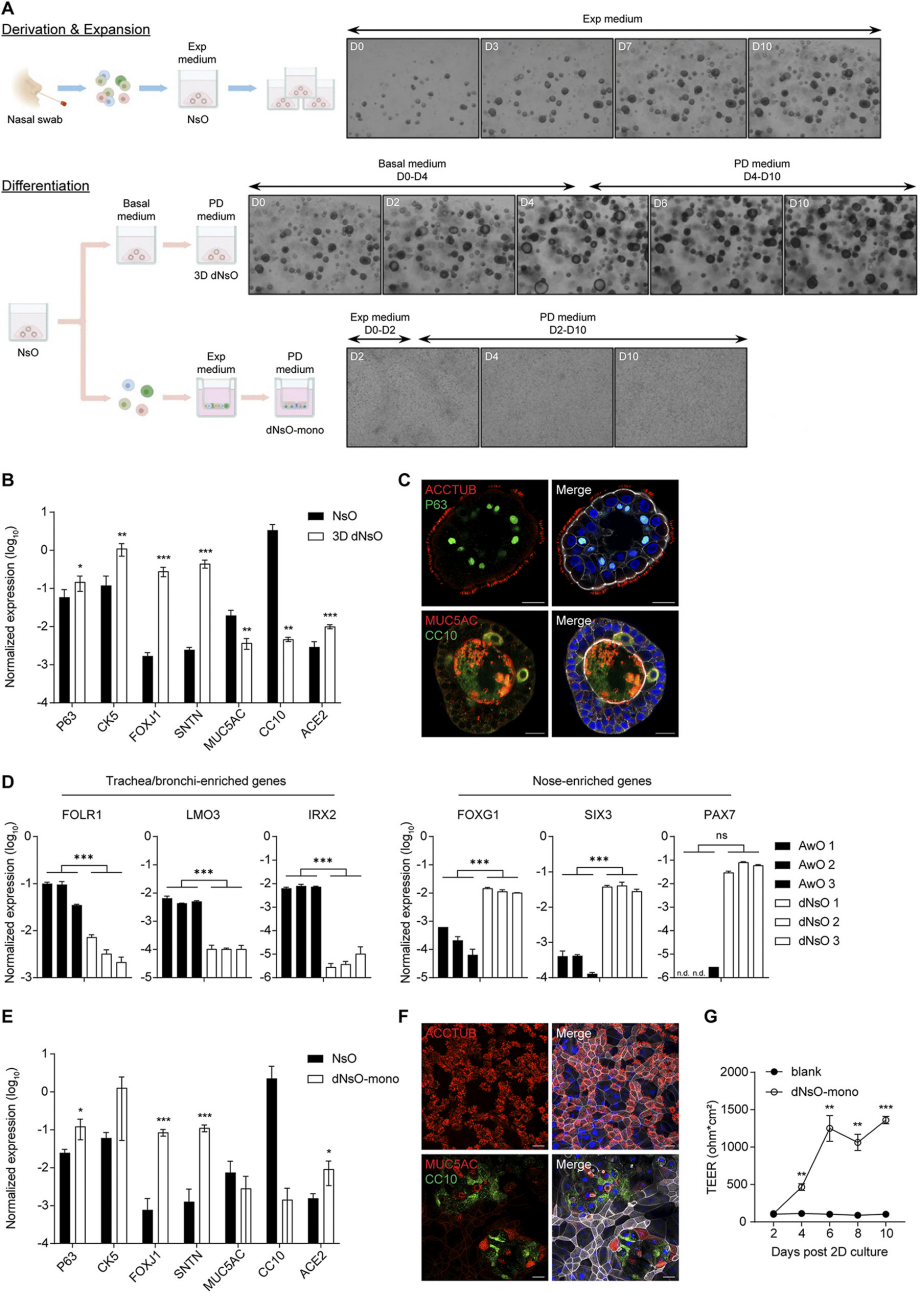
Establishment and characterization of human nasal organoids. (A) A schematic graph outlines the derivation, expansion, and differentiation of human nasal organoids. The upper panel shows the derivation of nasal organoids (NsO) from nasal cells. 3D undifferentiated nasal organoids undergo expansion in the expansion (Exp) medium. Photomicrographs show growing organoids on day 0 (D0), 3, 7, and 10 (magnification ×40). The bottom panel shows nasal organoids undergoing differentiation protocols to generate 3D differentiated nasal organoids (3D dNsO) or differentiated nasal organoid monolayer (dNsO-mono). Photomicrographs present differentiating 3D organoids on day 0, 2, 4, 6, and 10 in basal medium and PD medium sequentially (magnification ×40). Photomicrographs show differentiating organoid monolayers on day 2, 4, and 10 in expansion medium and PD medium sequentially (magnification ×100). (B) Parental 3D nasal organoids (NsO) and 3D differentiated nasal organoids (3D dNsO) were assessed for the expression level of cell-specific marker genes and SARS-CoV-2 receptor ACE2. Data represent the mean and s.d. of a representative experiment, *n* = 4. Two-tailed unpaired Student's *t* test. The experiment was independently performed in four organoid lines. (C) 3D dNsOs were subjected to immunofluorescence staining to label ACCTUB+ ciliated cells (top, red) and P63+ (top, green) basal cells, or MUC5AC+ goblet cells (bottom, red) and CC10+ (bottom, green) club cells. Nuclei and actin filaments were counterstained with DAPI (blue) and Phalloidin-647 (white), respectively. Scale bar, 20 μm. (D) Three lines of 3D differentiated human airway organoids (AwO) and other three lines of 3D differentiated nasal organoids (dNsO) were assessed for the expression levels of trachea/bronchi-enriched genes and nose-enriched genes. Data represent the mean and s.d., *n* = 2 in each of the organoid lines. Two-tailed unpaired Student's *t* test. (E) Parental 3D nasal organoids (NsO) and the differentiated nasal organoid monolayers (dNsO-mono) were assessed for the expression level of cell-specific marker genes and SARS-CoV-2 receptor ACE2. Data represent the mean and s.d. of a representative experiment, *n* = 4. Two-tailed unpaired Student's *t* test. The experiment was independently performed in two organoid lines. (F) Differentiated nasal organoid monolayers were subjected to immunofluorescence staining to label ACCTUB+ ciliated cells (top, red), or MUC5AC+ goblet cells (bottom, red) and CC10+ (bottom, green) club cells. Nuclei and actin filaments were counterstained with DAPI (blue) and Phalloidin-647 (white), respectively. Scale bar, 20 μm. (G) Trans-epithelial electrical resistance (TEER) of the dNsO-mono on the Transwell insert was measured every other day over 10 days. Blank insert without cell was measured as the baseline reading. Data represent the mean and s.d. of a representative experiment, *n* = 4. Two-tailed unpaired Student's *t* test. The experiment was independently performed in two organoid lines.

We collected nasal epithelial cells from the inferior turbinate of healthy donors using a flocked swab. The nasal epithelial cells were embedded in Matrigel and then overlaid with the organoid expansion medium supplemented with niche factors including R-spondin, Noggin, FGF7, and FGF10, the same medium for deriving human lung organoids (Fig. 1A). Cystic organoids emerged on day 2 or day 3 and became enlarged gradually. We consecutively passaged the nasal organoids every 10–12 days over 6 months. We have established more than 10 lines of nasal organoids from different donors with a 100% success rate, indicating the robustness and reproducibility of the protocol. Distinct from the lung organoids that always have a central lumen throughout expansion culture, nasal organoids normally develop a small central lumen in late days during each passage; most nasal organoids are solid cellular spheres devoid of a discernible central lumen.

The derived nasal organoids were propagated in the expansion medium in a 3-dimensional (3D) format by maintaining the clonogenic potential of adult stem cells or progenitor cells and directing the nasal organoids toward an immature state. To simulate the mature nasal epithelium, we tested an array of conditions and defined differentiation protocols to generate differentiated 3D organoids and organoid monolayers (Fig. 1A). Briefly, after 3D undifferentiated nasal organoids grew in the expansion medium for 10 days, we initiated differentiation with a 4-day incubation with a basal medium to enlarge the central lumen. It is an indispensable step since solid organoids without a central lumen may not develop adequate mucociliary differentiation; motile cilia were barely discernible in these solid organoids. Subsequently, we replaced the basal medium with a proximal differentiation (PD) medium, the same differentiation medium for airway organoid maturation (18), and further incubated for 6 days to generate fully differentiated nasal organoids. Hitherto, dense and motile cilia were readily discernible in every differentiated nasal organoid (Video S1). Similar to the human airway organoids and intestinal organoids, the organoids growing three-dimensionally in Matrigel normally develop an apical-in polarity since the extracellular matrix, herein provided by Matrigel, controls the epithelial polarity (24, 31).

Compared with the undifferentiated nasal organoids, the 3D differentiated nasal organoids exhibited a significant upregulation of cell type markers for basal cell (*P63*, *CK5*) and ciliated cell (*FOXJ1*, *SNTN*), as well as *ACE2*, the SARS-CoV-2 cellular receptor (Fig. 1B). The differentiated nasal organoids accommodated all airway epithelial cell types, i.e., ciliated, basal, goblet and club cells (Fig. 1C). Here, we deliberately display an apical-out nasal organoid to highlight the dense ACCTUB+ cilia. We also examined the expression of compartment-specific genes in 3D differentiated nasal organoids compared to that in 3D differentiated airway organoids derived from three different donors. Nose-enriched and trachea/bronchi-enriched genes were significantly higher in differentiated nasal and airway organoids, respectively (Fig. 1D), indicating that nasal organoids derived from nasal cells indeed retained the compartment-specific gene expression profile, consistent with the observations in native tracheobronchial and nasal epithelial cells as revealed by single-cell sequencing studies (6, 7).

The airway epithelium lining the human respiratory tract forms a physical barrier to protect against invading microbes, which is particularly important for the nasopharyngeal epithelium since it represents the very frontline to the external environment. To better model nasal epithelium exposure to invading pathogens, we generated differentiated nasal organoids of 2D format, organoid monolayers. Briefly, single cells dissociated from 3D undifferentiated nasal organoids were seeded onto Transwell inserts and incubated in the expansion medium for 2 days, which allowed cell attachment and expansion. We then switched to the PD medium and incubated for 8 days to generate confluent monolayers of differentiated nasal organoids. Similar to the 3D differentiated nasal organoids, basal cell (*P63*, *CK5*), ciliated cell (*FOXJ1*, *SNTN*) markers, and the *ACE2* receptor were significantly upregulated in differentiated nasal organoid monolayers (Fig. 1E). Four airway epithelial cell types are present in the organoid monolayers (Fig. 1F). Ciliated cells, the major cell population in the human nasal epithelium, are notably enriched in the organoid monolayers. Similar to the 3D counterparts, beating cilia were readily discernible under a microscope (Video S2). Trans-epithelial electrical resistance (TEER) is a reliable indication of epithelial integrity, the essential characteristic of a physical barrier. We measured TEER during the 10-day differentiation culture. As shown in Fig. 1G, TEER increased and reached a plateau on day 6. Thus, at the end of differentiation culture on day 10, the mature nasal organoid monolayers form an intact epithelial barrier on Transwell inserts. Collectively, we established expandable nasal organoids with a perfect success rate from readily accessible nasal epithelial cells. The expansion medium sustained the long-term expansion of nasal organoids by directing the organoids toward an immature state. The differentiation protocol enables the generation of differentiated nasal organoids of 3D and 2D format that faithfully simulate the nasal epithelium. These 3D differentiated nasal organoids and organoid monolayers of 2D format remain stable for around 2 weeks and 1 month, respectively, applicable to various experimental manipulations.

### Optimization of differentiated nasal organoid monolayers.

**FIG 2:**
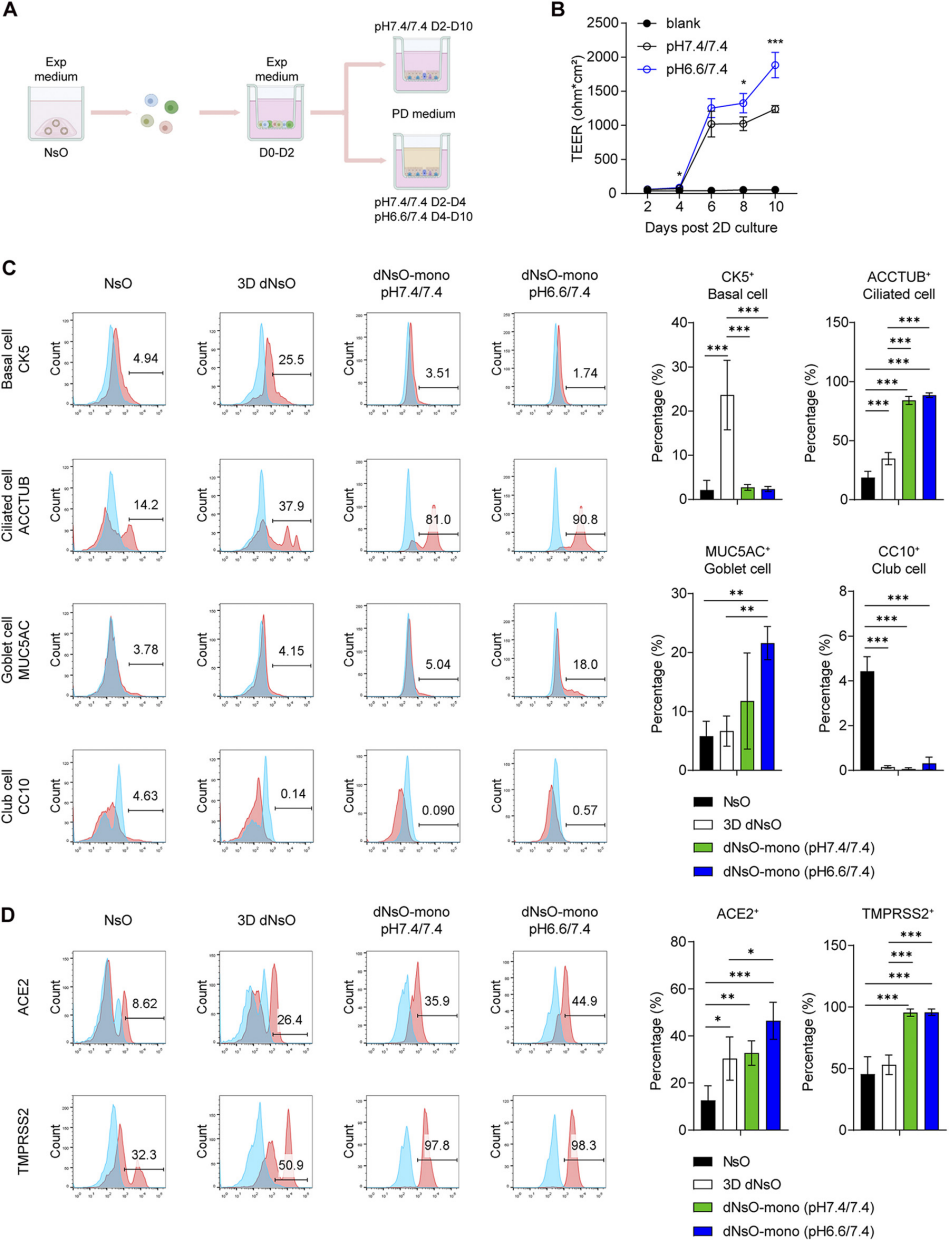
Optimization and characterization of differentiated nasal organoid monolayers. (A) A schematic graph outlines the generation of optimized differentiated nasal organoid monolayers (dNsO-mono). (B) Trans-epithelial electrical resistance (TEER) of the dNsO-mono differentiated at pH 7.4/7.4 or pH 6.6/7.4 PD medium were measured every other day over 10 days. A blank insert without any cell was measured as the baseline. Data represent the mean and s.d. of a representative experiment, *n* = 4. Two-tailed unpaired Student's *t* test. The experiment was independently performed in two organoid lines. (C) Parental 3D NsO, 3D dNsO, and dNsO-mono differentiated at pH 7.4/7.4 or pH 6.6/7.4 were applied to flow cytometry to examine the abundance of CK5+ basal cells, ACCTUB+ ciliated cells, MUC5AC+ goblet cells, and CC10+ club cells. Representative histograms are shown on the left. Red, cells stained with specific antibodies; blue, cells stained with isotype controls. Data on the right represent the mean and s.d. of a representative experiment, *n* = 4. Ordinary one-way ANOVA with Tukey's multiple comparison test. The experiment was independently performed in two organoid lines. (D) The organoids were applied to flow cytometry to examine the expression of ACE2 and TMPRSS2. Representative histograms are shown on the left. Red, cells stained with specific antibodies; blue, cells stained with isotype controls. Data on the right represent the mean and s.d. of a representative experiment, *n* = 4. Ordinary one-way ANOVA with Tukey's multiple comparison test. The experiment was independently performed in two organoid lines.

The surface liquid in the human airways, including nasal mucosa, is slightly acidic with an average pH of 6.6 (32). Moreover, the low pH of airway surface liquid is one of the determinants for SARS-CoV-2 active infection (33, 34). However, in the differentiation culture of organoid monolayers as described above, the proximal differentiation (PD) media in both top and bottom chambers were buffered with HEPES at a pH of 7.4, the physiological pH of interstitial fluid. We speculated that a slightly acidic medium in the top chamber and a physiological alkaline medium in the bottom chamber might better simulate the *in vivo* milieu and generate more physiological-relevant nasal organoids. To this end, after the cells reached confluence on Transwell inserts, we replaced HEPES buffer in the top medium (i.e., the PD medium) with a PIPES buffer to create a pH of 6.6, while the original HEPES remained in the bottom medium with a pH of 7.4. Namely, organoid monolayers were incubated in the modified differentiation medium with a pH of 6.6/7.4 (top/bottom) or the original differentiation medium with a pH of 7.4/7.4 (Fig. 2A). The nasal organoids incubated in the differentiation medium at pH 6.6/7.4 displayed a higher TEER than those at pH 7.4/7.4, indicating the formation of a strengthened epithelial barrier (Fig. 2B).

We then examined the cellular composition and ACE2 and TMPRSS2 in all the differentiated nasal organoids, including 3D differentiated organoids and organoid monolayers undergoing differentiation at pH 6.6/7.4 or pH 7.4/7.4, in comparison with those in the 3D undifferentiated nasal organoids. We found that ciliated cells represented the major cell population in nasal organoid monolayers. Goblet cells were significantly enriched in nasal organoid monolayers at pH 6.6/7.4 than those at pH 7.4/7.4 and 3D differentiated nasal organoids (Fig. 2C). Interestingly, the proportion of basal cells was the highest in 3D differentiated nasal organoids. In contrast to ciliated cells, club cells significantly decreased after differentiation culture. Of note, the differentiation procedure increased ACE2+ cells in all forms of organoids; the percentage of ACE2+ cells was the highest in nasal organoid monolayers at pH 6.6/7.4. In addition, TMPRSS2+ cells were significantly more abundant in organoid monolayers than both differentiated and undifferentiated 3D organoids (Fig. 2D). Collectively, among all the differentiated organoids, nasal organoid monolayers differentiated in pH 6.6/7.4 most adequately phenocopy the native nasal epithelium for modeling SARS-CoV-2 infection.

### SARS-CoV-2 replication and higher replicative fitness of emerging variants in nasal organoids.

**FIG 3:**
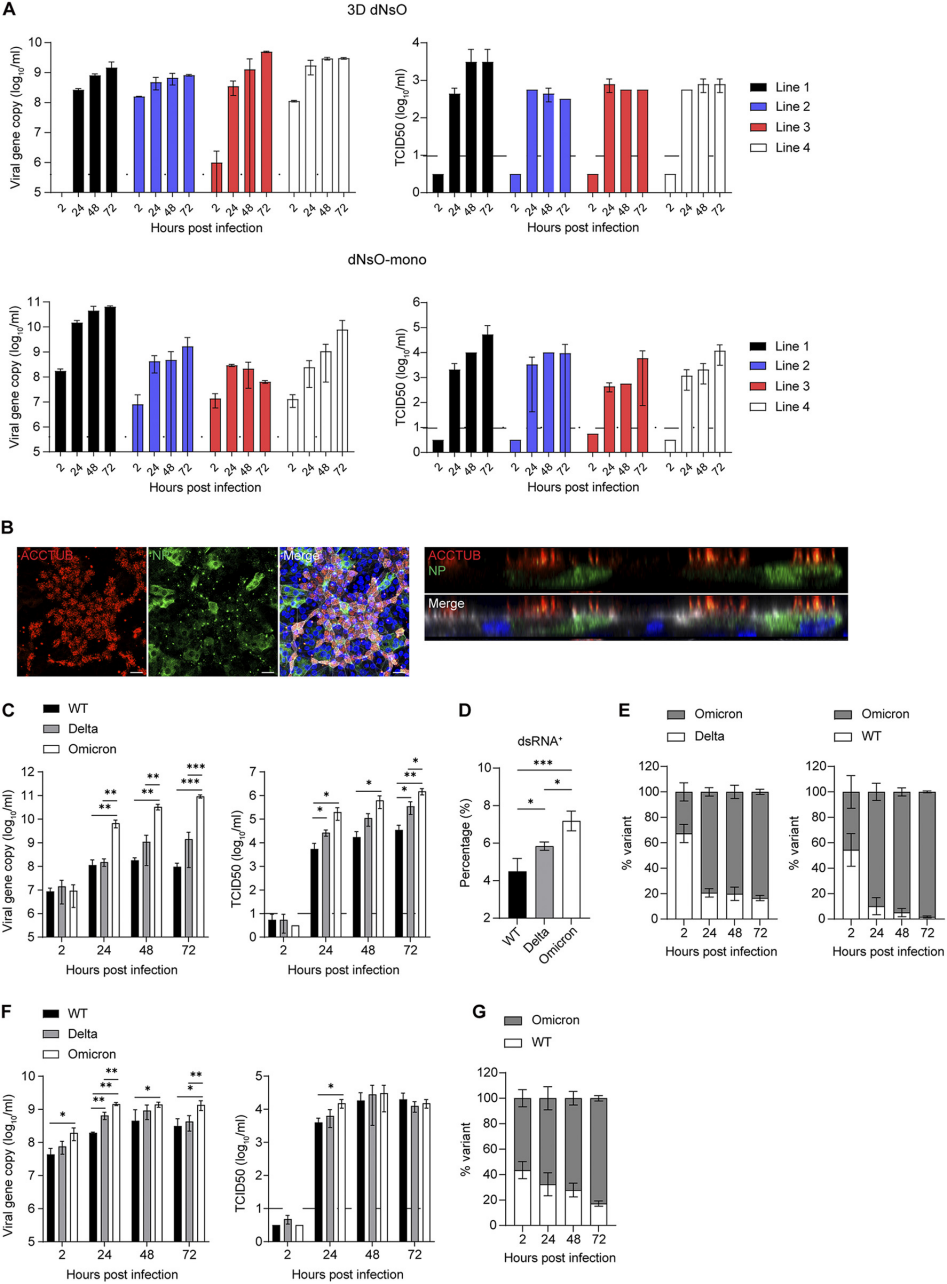
SARS-CoV-2 infection and replicative fitness in differentiated nasal organoids. (A) At the indicated hours postinoculation, culture media were harvested from four lines of 3D dNsO (top, 1 MOI) or dNsO-mono (bottom, 0.1 MOI) infected with SARS-CoV-2 and applied to viral load detection of RdRp gene by RT-qPCR and viral titration by TCID50 assay. Data represent mean and s.d., *n* = 2 in each of the organoid lines. The dashed line indicates the detection limit. (B) At 24 h postinoculation (5 MOI), SARS-CoV-2 infected dNsO-mono were co-stained with α-NP (green) and α-ACCTUB (red). Confocal images of en face (top) and cross-section (bottom) are shown. Nuclei and actin filaments were counterstained with DAPI (blue) and Phalloidin-647 (white). Scale bar, 20 μm. (C) At the indicated hours postinoculation with wildtype (WT), Delta, or Omicron variant (0.1 MOI), culture media were harvested from the top chambers of the dNsO-mono and applied to viral load detection and viral titration. Data show mean and s.d. of a representative experiment, *n* = 3. Multiple *unpaired t test* with multiple comparisons using Holm-Sidak method. The experiment was independently performed in two organoid lines. (D) At 24 h postinoculation (1 MOI), SARS-CoV-2 WT, Delta, or Omicron infected dNsO-mono were dissociated and applied to flow cytometry to detect dsRNA+ cells. Data represent the mean and s.d. of a representative experiment, *n* = 4. Ordinary one-way ANOVA with Tukey's multiple comparison test. The experiment was independently performed in two organoid lines. (E) At the indicated hours postinoculation with 1:1 mixture of Omicron and Delta (left), or mixture of Omicron and WT viruses (right) (total 0.1 MOI), culture media were harvested from the top chambers of the dNsO-mono and applied to viral load detection using variant specific primers and probes. Data represent the mean and s.d. of a representative experiment, *n* = 3. (F) At the indicated hours postinoculation with wildtype (WT), Delta, or Omicron variant (0.1 MOI), culture media were harvested from the apical chambers of the airway organoid monolayer (AwO-mono) and applied to viral load detection and viral titration. Data show the mean and s.d. of a representative experiment, *n* = 3. Multiple *unpaired t test* with multiple comparisons using Holm-Sidak method. (G) At the indicated hours postinoculation with 1:1 mixture of Omicron and WT (total 0.1 MOI), culture media were harvested from the apical chambers of the AwO-mono and applied to viral load detection using variant-specific primers and probes. Data represent the mean and s.d. of a representative experiment, *n* = 3.

We next examined SARS-CoV-2 replication kinetics in differentiated nasal organoids derived from four different donors. Both differentiated 3D nasal organoids and organoid monolayers sustained SARS-CoV-2 replication, as shown by increased viral RdRp gene copy number and infectious titer (Fig. 3A), although organoids derived from different donors showed variable replication capacity. The replicative fitness of SARS-CoV-2 was higher in organoid monolayers than in the 3D counterparts, which might be ascribed to the higher ACE2 and TMPRSS2 expression in the former than the latter, as shown in Fig. 2D. Immunostaining showed many SARS-CoV-2-infected cells in nasal organoid monolayers; viral NP co-localizing or not co-localizing with ACCTUB+ ciliated cells in both en face and cross-sectional images (Fig. 3B).

The Delta and Omicron variants overtook the prior circulating strains after the emergence. To elucidate virus-host interaction underlying their enhanced transmissibility over the ancestral viruses, we inoculated an ancestral wildtype (WT) strain, a Delta strain and an Omicron strain onto the nasal organoid monolayers induced differentiation at pH 6.6/7.4 since these differentiated nasal organoids best model SARS-CoV-2 infection. Quantification of viral load in the culture medium and viral titration revealed the higher replicative fitness of the Omicron variant than the Delta variant, followed by the WT strain (Fig. 3C). The Omicron variant showed a significantly higher viral titer of around 2 log units than the WT strain. We examined the infection rate, i.e., the percentage of virus-infected cells 24 h after a high MOI inoculation. Flow cytometry analysis demonstrated significantly higher infectivity of the Omicron variant than the Delta variant; the lowest infection rate was found in the WT strain (Fig. 3D).

We then performed a pairwise competition assay in the nasal organoid monolayers. The Omicron variant quickly outgrew the Delta variant (Fig. 3E, left). The replicative advantage of the Omicron variant was even more dramatic when it was co-inoculated with the WT strain (Fig. 3E, right). We also compared the replicative fitness of three virus strains in differentiated airway organoid monolayers. The airway organoids reproduced a similar order of replicative capacity of these three virus strains, yet the Omicron's replicative advantage was less remarkable than that in the nasal organoid monolayers (Fig. 3F). The higher replication capacity of the Omicron variant than the WT virus was verified in the airway organoids by the competition assay. Similar to the replication data, the outgrowth of the Omicron variant was not as prominent as that in nasal organoids (Fig. 3G). In contrast, replication kinetics and competition assays in Vero E6/TMPRSS2 cells revealed a lower replicative fitness of Delta and Omicron variants than the WT strain (Fig. S1A and B and Text S1 in the supplemental material), which is contradictory to the higher infectivity and transmissibility of these variants observed in humans.

10.1128/mbio.01944-22.1 FIG S1 (A) At the indicated hours post-inoculation with wildtype (WT), Delta, or Omicron variant (0.01 MOI), culture media were harvested from the Vero E6/TMPRSS2 cells and applied to viral load detection and viral titration. Data show the mean and s.d. of a representative experiment, *n* = 3. Multiple unpaired t-test with multiple comparisons using Holm-Sidak method. The experiment was independently performed in two organoid lines. (B) At the indicated hours post-inoculation with a 1:1 mixture of Omicron and Delta (left), or a mixture of Omicron and WT viruses (right) (total 0.01 MOI), culture media were harvested from the Vero E6/TMPRSS2 cells and applied to viral load detection using variant-specific primers and probes. Data represent the mean and s.d. of a representative experiment, *n* = 3. The experiment was independently performed in two organoid lines. Download FIG S1, TIF file, 0.5 MB. Copyright © 2022 Chiu et al. 2022 Chiu et al. https://creativecommons.org/licenses/by/4.0/ This content is distributed under the terms of the Creative Commons Attribution 4.0 International license.

10.1128/mbio.01944-22.1 TEXT S1 Supplemental material legends. Download Text S1, DOCX file, 0.03MB. Copyright © 2022 Chiu et al. 2022 Chiu et al. https://creativecommons.org/licenses/by/4.0/ This content is distributed under the terms of the Creative Commons Attribution 4.0 International license.

Overall, the three virus strains exhibited differential replicative fitness and infectivity in differentiated nasal organoid monolayers, which accurately reproduced the Omicron's highest transmissibility as observed in real-life epidemiological observations. In addition, nasal organoids recapitulated the variable transmissibility more adequately than airway organoids.

### Preferential infection of ciliated cells by SARS-CoV-2.

**FIG 4:**
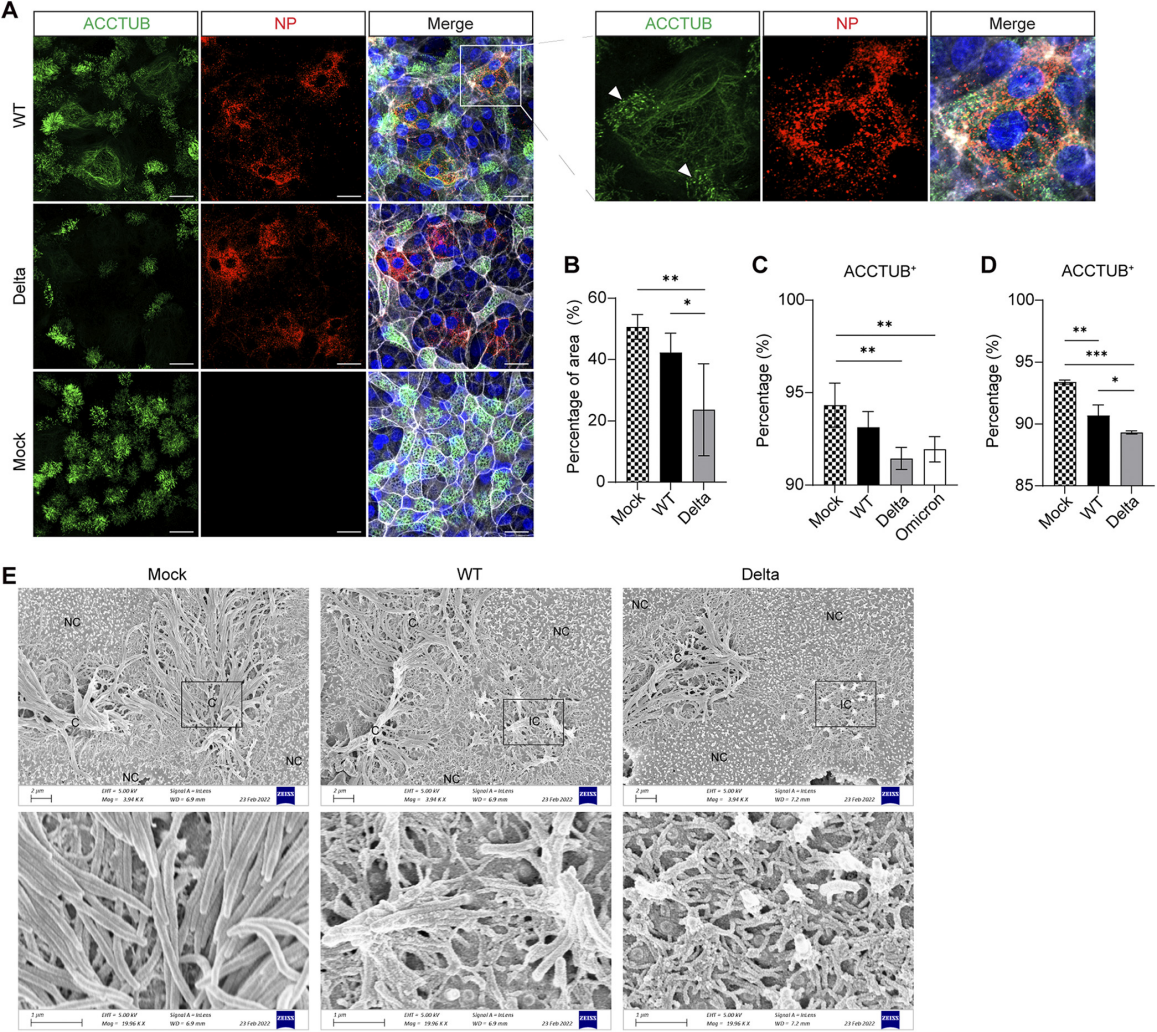
SARS-CoV-2 targets ciliated cells. (A) At 24 h postinoculation (1 MOI), WT-, or Delta-infected or mock-infected dNsO-mono were co-stained with α-NP (red) and α-ACCTUB (green). Nuclei and actin filaments were counterstained with DAPI (blue) and Phalloidin-647 (white). Scale bar, 20 μm. An enlarged image of the boxed area is shown on the right. Arrows indicate the remaining cilia on an infected ciliated cell. (B) Image quantification of the immunofluorescence staining of mock-, WT-, or Delta-infected dNsO-mono. The percentage of area covered by ACCTUB labeled cilia was shown. Data represent the mean and s.d. of a representative experiment, *n* = 5. Ordinary one-way ANOVA with Tukey's multiple comparison test. (C) At 24 h postinoculation (1 MOI), mock-, WT-, Delta-, or Omicron-infected dNsO-mono were dissociated and applied to flow cytometry to detect ACCTUB+ ciliated cells. Data represent the mean and s.d. of a representative experiment, *n* = 4. Ordinary one-way ANOVA with Tukey's multiple comparison test. The experiment was independently performed in two organoid lines. (D) At 24 h postinoculation (1 MOI), mock-, WT-, or Delta -infected airway organoid monolayer (AwO-mono) were dissociated and applied to flow cytometry to detect ACCTUB+ ciliated cells. Data represent the mean and s.d. of a representative experiment, *n* = 3. Ordinary one-way ANOVA with Tukey's multiple comparison test. The experiment was independently performed in two organoid lines. (E) At 24 h postinoculation (1 MOI), mock-, WT-, or Delta-infected dNsO-mono were applied to scanning electron microscopy. NC, non-ciliated cell; C, ciliated cell; IC, infected ciliated cell. The enlarged images of the box areas are shown at the bottom.

We examined the infected nasal organoid monolayers by confocal imaging to elucidate the cellular pathology of SARS-CoV-2 infection. Ciliated cells are distributed evenly in the mock-infected nasal organoid monolayers, with dense ACCTUB+ cilia on the apex of ciliated cells (Fig. 4A). We noted ciliary damage and loss of ciliated cells in SARS-CoV-2 infected nasal organoids, consistent with the observations reported in cultured human airway epithelial cells (11). The dense hairy cilia on the apex of normal ciliated cells were disrupted and/or deformed into a fibrous clump (Fig. 4A). In the organoids infected by the WT virus (boxed), two small clusters of cilia remained on a ciliated cell (white arrows), whereas most cilia were destroyed. Ciliary damage was more prominent in organoids infected by the Delta variant than those infected by WT strain. Image analysis in 5 randomly selected frames revealed a significantly decreased proportion of ACCTUB+ cells in the virus-infected organoids, especially those infected with the Delta variant (Fig. 4B). Flow cytometry analysis verified the results of image analysis. While WT virus infection modestly depleted ACCTUB+ ciliated cells in nasal organoid monolayers, the infection of the Delta variant or the Omicron resulted in a further and significant depletion (Fig. 4C). Consistently, flow cytometry analysis of the airway organoid monolayers infected by the WT and Delta variant showed similar results (Fig. 4D). We performed scanning electron microscopy to examine the cytopathological changes in more details. In the uninfected nasal organoids, we visualized dense cilia with a smooth surface on the apex of ciliated cells (Fig. 4E). However, cilia became shorter, tangled, and deformed in the organoids infected by the WT strain and the Delta variant (Fig. 4E). Thus, SARS-CoV-2 preferentially infected ciliated cells in nasal organoids and airway organoids, causing ciliary damage and loss.

### SARS-CoV-2 infection disrupting cell adhesion to enhance virus transmission.

**FIG 5:**
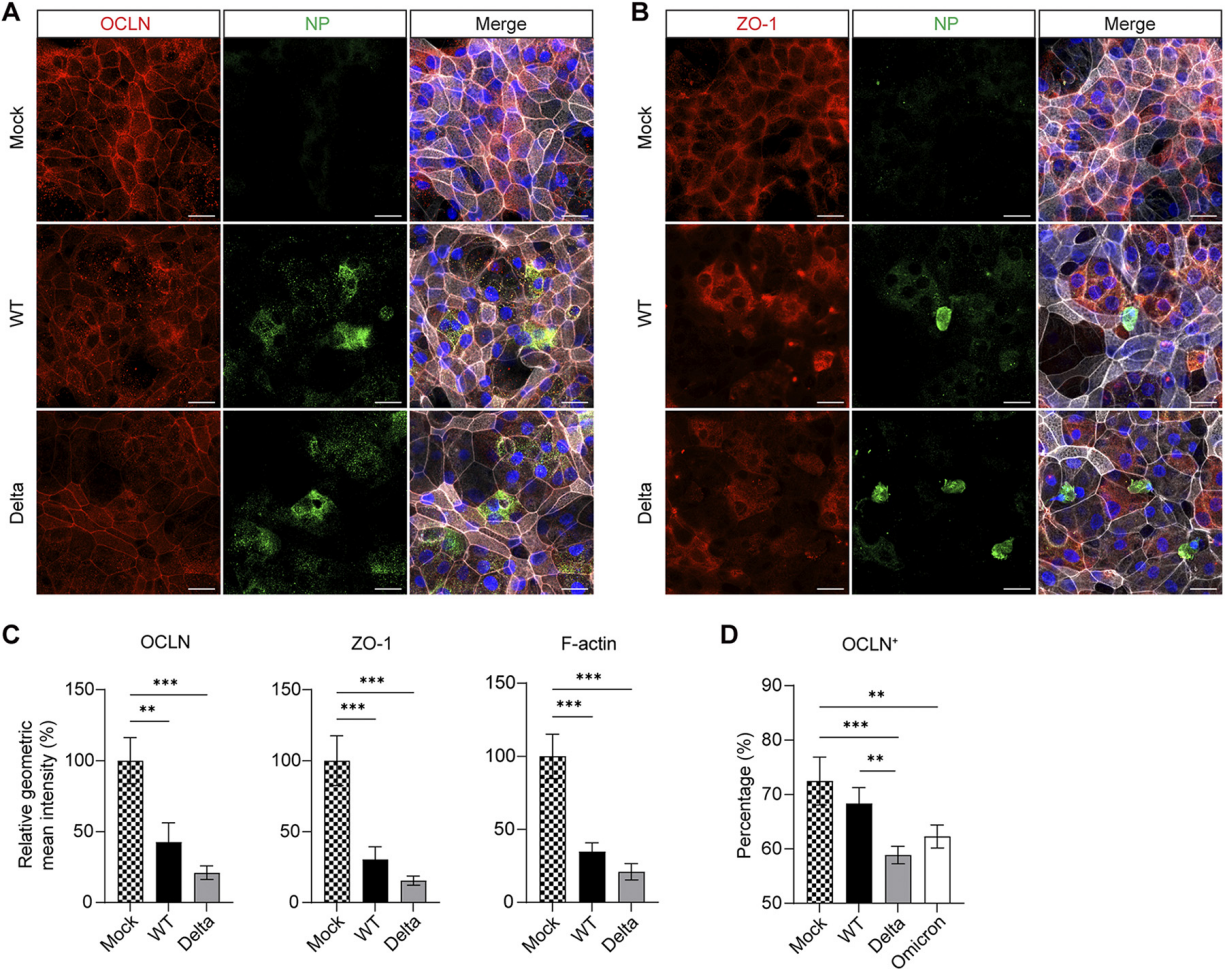
SARS-CoV-2 damages cellular junction. At 24 h postinoculation (1 MOI), mock-, WT-, or Delta-infected dNsO-mono were co-stained with (A) α-NP (green) and α-OCLN (red), or (B) α-NP (green) and α-ZO-1 (red). Nuclei and actin filaments were counterstained with DAPI (blue) and Phalloidin-647 (white). Scale bar, 20 μm. (C) Image quantification of the immunofluorescence labeled mock-, WT-, or Delta-infected dNsO-mono. Geometric mean intensity relative to OCLN (left), ZO-1 (middle), and F-actin (right) in mock-infected organoids is shown. Data represent the mean and s.d. of a representative experiment, *n* = 3. Ordinary one-way ANOVA with Tukey's multiple comparison test. (D) At 24 h postinoculation (1 MOI), mock-, WT-, Delta-, or Omicron-infected dNsO-mono were dissociated and applied to flow cytometry to detect OCLN+ cells. Data represent the mean and s.d. of a representative experiment, *n* = 4. Ordinary one-way ANOVA with Tukey's multiple comparison test. The experiment was independently performed in two organoid lines.

Apart from the mucociliary clearance afforded by ciliated and goblet cells, the formation of tight junctions between adjacent epithelial cells represents a subcellular level of host defense to protect against invading pathogens and maintain the homeostasis of the human respiratory tract. Meanwhile, viruses have developed multiple mechanisms to disassemble tight junctions to facilitate infection or exploit specific cell junction targets for cellular entry (35). Zonula Occludens (ZO) family link tight junction proteins such as Occludin and Claudins to the actin cytoskeleton. As shown in Fig. 5A and B, Occludin (OCLN) and ZO-1 proteins delineate the outline of each cell in mock-infected nasal organoid monolayers, indicating the formation of intact tight junctions by these adhesion molecules. We observed altered cell junctions in the organoids infected with the WT and Delta viruses. In the organoids infected by WT strain, ZO-1 and OCLN diffused or diminished in the area within or near the virus-infected cells, whereas Delta variant infection caused a widespread disassemble of cellular junction throughout the monolayers, not limited to the infected cells, suggesting a possible paracrine effect. We quantified the signal intensity in 3 randomly selected frames and found a significantly eliminated OCLN and ZO-1 in WT virus-infected nasal organoids compared to mock-infected organoids. The organoids inoculated with the Delta variant exhibited a further diminished ZO-1, OCLN and cytoskeleton F-actin than those infected by the WT virus (Fig. 5C). Flow cytometry analysis verified SARS-CoV-2 induced disruption of OCLN, and a more disruptive effect of the Delta and Omicron variants in nasal organoid monolayers (Fig. 5D). The destructive effect of SARS-CoV-2 infection on cellular junction was further verified in airway organoid monolayers by immunostaining (Fig. S2A to B) and flow cytometry analysis (Fig. S2C in the supplemental material). Thus, SARS-CoV-2 disrupted tight junctions in nasal organoids and airway organoids, breaching the epithelial barrier.

10.1128/mbio.01944-22.2 FIG S2 (A) At 24 h post-inoculation (1 MOI), mock-, WT-, or Delta-infected airway organoid monolayer (AwO-mono) were co-stained with α-OCLN (red) and α-ZO-1(green). Nuclei and actin filaments were counterstained with DAPI (blue) and Phalloidin-647 (white). Scale bar, 20 μm. (B) Image quantification of the immunofluorescence labeled mock-, WT-, or Delta-infected AwO-mono. Geometric mean intensity relative to mock of OCLN (left), ZO-1 (middle), and F-actin (right) were shown. Data represent the mean and s.d. of a representative experiment, *n* = 3. Ordinary one-way ANOVA with Tukey's multiple comparison test. (C) At 24 h post-inoculation (1 MOI), mock-, WT-, or Delta-infected AwO-mono were dissociated and applied to flow cytometry to detect OCLN+ cells. Data represent the mean and s.d. of a representative experiment, *n* = 3. Ordinary one-way ANOVA with Tukey's multiple comparison test. The experiment was independently performed in two organoid lines. Download FIG S2, TIF file, 2.7 MB. Copyright © 2022 Chiu et al. 2022 Chiu et al. https://creativecommons.org/licenses/by/4.0/ This content is distributed under the terms of the Creative Commons Attribution 4.0 International license.

## DISCUSSION

The mucosal epithelium lining the nasopharyngeal compartment is the entry portal and primary target of respiratory pathogens, including SARS-CoV-2; meanwhile nasal epithelial cells express the highest level of SARS-CoV-2 entry factors among multiple interrogated human tissues (36). High susceptibility of nasal epithelial cells to SARS-CoV-2, robust viral replication in these cells and subsequent viral shedding constitute the biological basis underlying viral pathogenesis and its high transmissibility. As such, it is imperative to establish an *in vitro* model of the nasal epithelium to elucidate SARS-CoV-2 infection and virus-host interaction. In this study, we established and characterized a robust organoid culture system of the human nasal epithelium to model SARS-CoV-2 upper respiratory infection. It is a two-phase culture system, that is, expansion and differentiation culture (Fig. 1A). The expansion medium sustained long-term expansion of nasal organoids for more than 6 months by directing the 3D undifferentiated organoids toward an immature state. We defined the differentiation protocols to generate differentiated 3D nasal organoids and organoid monolayers (Fig. 1B, C, E, and F). The differentiated nasal organoids adequately simulate the native nasal epithelium. Namely, the nasal organoid culture system enables a highly efficient reconstruction and stable expansion of the human nasal epithelium in culture plates for various experimental applications.

We further optimized the differentiation condition and generated highly differentiated nasal organoid monolayers by mimicking the slightly acidic surface fluid on the nasal mucosa (Fig. 2A). Among all the differentiated forms of nasal organoids, organoid monolayers induced differentiation with the slightly acidic medium (pH 6.6) in the top chamber displayed a more intensified epithelial barrier (Fig. 2B) and a higher expression of ACE2, the cellular receptor for SARS-CoV-2 infection (Fig. 2D). Notably, the optimized nasal organoid monolayers accurately reproduce the higher infectivity and elevated replicative fitness of the Omicron variant than prior circulating variants by flow cytometry analysis of infection rate, viral titration detection of viral propagation and the competition assay (Fig. 3C–E).

The derivation, expansion and differentiation of nasal organoids are based on our prior experience of deriving long-term expandable lung organoids and generating airway organoids with modifications and optimization. A significant advance is the cell source for deriving nasal organoids. We procured nasal epithelial cells from the inferior turbinate of healthy donors using a flocked swab, which is a noninvasive and readily conductable procedure, unlike deriving lung organoids using cells from bronchoalveolar lavage or resected tissues that require the invasive manipulations of physicians and surgeons. More importantly, prior studies of human intestinal organoids, the first adult stem cell-derived organoid model, revealed that the epithelial organoids generated from different intestinal segments retain the structural and functional characteristics from which they are derived (29), because tissue identity is imprinted in the adult stem cells and maintained in these organoids during long-term expansion culture (37). Thus, nasal epithelial cells would be an optimal source for deriving physiologically-relevant nasal organoids. Indeed, nasal organoids exhibited a compartment-specific gene expression profile (Fig. 1D). More importantly, nasal organoids retain the genotypic and phenotypic characteristics of original cells, which allows the generation of personalized nasal organoids and provides a facile and robust *in vitro* model of upper respiratory epithelium for various applications of personalized medicine.

Interestingly, nasal organoids have been derived with a perfect success rate, which was beyond our expectation. Shortly after, we figured out the reason for this superior efficiency from a recent single-cell RNA sequencing study, in which the authors characterized the cells harvested from healthy individuals and COVID-19 patients via nasal brushing and found that 60% were ciliated and goblet cells, suprabasal and club cells accounted for 30% approximately (5). Suprabasal cell is an intermediate cell population between basal and club cells (6). Basal/suprabasal and club cells were reported to be progenitor cells for respiratory epithelial regeneration (38–40). These progenitor cells have clonal expansion capacity *in vitro* and proliferate to form organoids (40–42). In this study, the expansion medium supplemented with R-spondin, Noggin and FGF sustains the clonogenicity of these progenitor cells and enables a consecutive expansion of undifferentiated nasal organoids for approximately 6 months, albeit with a shorter period compared to tissue-derived lung organoids that are expandable for over 1 year. The results implicate that, in terms of clonogenic potential, the progenitor cells or adult stem cells residing in human lung tissues may outperform the cells procured from nasal mucosa noninvasively. Nonetheless, the facile cell procurement procedure circumvented the relatively shorter expandability of nasal organoids, highlighting nasal organoids as a robust and personalized *in vitro* model of the nasal epithelium. Notably, in the nasal organoids, the Omicron variant exhibited a significantly higher replicative fitness and infection rate than the earlier circulating variants and outgrew the latter in a competition assay, indicating nasal organoids able to recapitulate the infectivity of emerging SARS-CoV-2 variants (Fig. 3C–E). In contrast, the Omicron and Delta variants exhibited lower replicative fitness than the WT strain in Vero E6/TMPRSS2 cells (Fig. S1A and B), which was repeatedly demonstrated in other studies using cell lines (43), highlighting the strength of biologically relevant nasal organoids.

In Fig. 3B, it seems that SARS-CoV-2 targeted both ciliated cells and non-ciliated cells. The subsequent interrogations gave us a rational interpretation that those infected “non-ciliated” cells, at least part of them, result from ciliary damage and/or depletion mediated by the virus infection (Fig. 4A–E). SARS-CoV-2 cellular tropism in ciliated cells, the resultant ciliary damage and depletion of ciliated cells were documented in SARS-CoV-2 infection in cultured primary nasal and airway epithelial cells and single-cell RNA sequencing studies of COVID-19 patients (4, 5, 10, 11, 13, 14). Ciliated cells are the primary target of SARS-CoV-2. Accordingly, the higher replicative fitness of the Omicron and Delta variants in nasal organoid monolayers led to a more prominent depletion of the ciliated cell (Fig. 4C). Motile cilium-driven mucociliary clearance represents an important host defense against pathogens. Ciliary damage caused by SARS-CoV-2 would inevitably compromise the mucociliary clearance, leading to exaggerated tissue pathology. Cellular adhesion, including tight junction, represents an additional layer of host defense to protect against invading pathogens. We demonstrated that the more infective Omicron and Delta variants disrupted tight junctions more severely than the earlier variant through imaging analysis and quantitative flow cytometry analysis (Fig. 5A to D and S2A to C). Collectively, SARS-CoV-2 viruses, especially the more transmissible variants, destroy ciliated cells leading to a compromised mucociliary clearance, and disassemble tight junctions to breach the epithelial barrier. These pathological changes render the uninfected cells more vulnerable, thereby facilitating virus spread and transmission.

When the study was approaching the end, Rajan et al. recently reported a human “nose organoid” model (44). Interestingly, the authors also established nose organoids based on our reported protocol of generating human airway organoids (18, 19). However, the expandability of these nose organoids was not clearly defined. The nose organoids were only differentiated using air-liquid interface culture and characterized by qualitative assays such as H&E staining and IF staining.

Herein, we established a robust nasal organoid culture system and fully characterized the organoids. The nasal organoids are stably passaged for more than 6 months, providing a renewable source of nasal epithelial cells. We moved forward and further optimized the nasal organoid monolayers by mimicking the native microenvironment of the nasal epithelium. We demonstrated that the optimized nasal organoid monolayers adequately recapitulate SARS-CoV-2 high infectivity, particularly the highly transmissible Delta and Omicron variants, in the upper respiratory tract. These optimized nasal organoid monolayers are superior to most, if not all, existing respiratory organoid models, including our previously established airway organoid monolayers. A limitation of our nasal organoid culture system is that we have not characterized the progenitor cells giving rise to nasal organoids. Nonetheless, we establish a robust organoid system enabling stable expansion and reconstruction of the human nasal epithelium in culture plates. The nasal organoid culture system provides an unlimited source of physiologically-active nasal epithelial cells for studying respiratory pathogens, circumventing the restriction of cultured primary nasal epithelial cells. More importantly, the facile procedure of procuring nasal cells and a zero-failure establishment efficiency allow researchers to establish personalized nasal organoids readily, which will pave a new avenue for many exciting organoid-based investigations for combating the COVID-19 pandemic.

## MATERIALS AND METHODS

### Establishment, expansion, and differentiation of nasal organoids.

The study has been approved by the Institutional Review Board of the University of Hong Kong/Hospital Authority Hong Kong West Cluster (UW21-695). Ten lines of human nasal organoids (NsO) were established using the freshly isolated nasal epithelial cells from nine healthy donors. Briefly, nasal cells were collected from the nasal turbinate regions using the specimen collection flocked swab (iClean, Biomed Diagnostics). After washing and digestion with 10X TrypLE Select (Gibco) for 5 min at 37°C, the resultant single cells were embedded in 70% Matrigel and seeded into a 24-well suspension culture plate. After solidification, droplets were incubated in the expansion medium (Table S1 in the supplemental material) at 37°C in a humidified incubator with 5% CO_2_. The expansion medium was replenished every other day; the 3D undifferentiated organoids were passaged every 1 to 2 weeks with a ratio between 1:3 to 1:10, depending on whether mechanical shearing or enzymatic digestion was used to split the organoids (21).

10.1128/mbio.01944-22.5 TABLE S1 Composition of the expansion medium. Download Table S1, XLSX file, 0.01 MB. Copyright © 2022 Chiu et al. 2022 Chiu et al. https://creativecommons.org/licenses/by/4.0/ This content is distributed under the terms of the Creative Commons Attribution 4.0 International license.

To induce the maturation of nasal organoids into 3D differentiated nasal organoids (3D dNsO), we first incubate the 3D nasal organoids in a basal medium (Advanced DMEM/F-12 (Gibco) supplemented with 1% HEPES, 1% GlutaMAX and 1% Penicillin/Streptomycin) for 4 days, followed by incubation with the PD medium (PneumaCult-ALI medium (STEMCELL Technologies) supplemented with 10 μM DAPT) for 6–10 days. The medium was replenished every other day. To induce differentiation into differentiated nasal organoid monolayers (dNsO-mono), we treated 3D nasal organoids with 10X TrypLE Select (Invitrogen) for 5 min at 37°C and seeded the single cells onto Transwell inserts at a density of approximately 4 × 10^5^ cells per cm^2^. The cells were cultured in the expansion medium for 1 to 2 days, then changed to the PD medium and incubated for another 8–12 days. The PD medium was supplied to both the top and bottom chambers and was replenished every other day. To optimize organoid monolayer differentiation, after the cells reached complete confluence in the PD medium in 1 to 2 days, we changed the media in the top chambers to the PD medium buffered with PIPES (Sigma-Aldrich) to create a pH around 6.6, while those in the bottom chambers remained buffered with HEPES (Gibco) to maintain a pH around 7.4. Organoids were incubated in these buffered PD media for 6–10 days to achieve maturation. A Millicell ERS-2 Volt-Ohm Meter (EMD Millipore) was used to measure the trans-epithelial electrical resistance (TEER) of organoid monolayers every other day to assess the epithelial barrier integrity.

The 3D undifferentiated nasal organoids, 3D differentiated nasal organoids, and nasal organoid monolayers were harvested and applied to RNA extraction using MiniBEST Universal RNA Extraction kit (TaKaRa), followed by reverse transcription using Transcriptor First Strand cDNA Synthesis Kit (Roche) and oligo(dT) primer. The resultant cDNAs were used to measure mRNA expression levels of cellular genes (Table S2 in the supplemental material) using the LightCycler 480 SYBR green I Master Mix (Roche) as described previously (45). Photomicrographs and videos of the organoids were acquired using Nikon Eclipse TS100 Inverted Routine Microscope.

10.1128/mbio.01944-22.6 TABLE S2 qPCR primer list. Download Table S2, XLSX file, 0.01 MB. Copyright © 2022 Chiu et al. 2022 Chiu et al. https://creativecommons.org/licenses/by/4.0/ This content is distributed under the terms of the Creative Commons Attribution 4.0 International license.

### Virus infection and detection.

SARS-CoV-2 isolate HKU-001a (WT, GenBank accession number MT230904), Delta variant (B.1.617.2; GenBank OM212471), and Omicron variant (B.1.1.529; GenBank OM212473) were propagated in Vero E6/TMPRSS2 cells (JCRB1819) purchased from JCRB cell bank and titrated with plaque assay as we described previously (46, 47). We always use differentiated organoids for infection experiments. 3D nasal organoids were sheared mechanically using a glass Pasteur pipette and incubated with the virus for 2 h at 37°C. The inoculated organoids were re-embedded into Matrigel and then incubated in the basal medium.

After washing twice with the basal medium, nasal organoid monolayers were apically incubated with the virus for 2 h at 37°C, followed by incubation in the basal medium. To assess replication kinetics, after inoculation in organoid monolayers with an MOI of 0.1 or inoculation in 3D organoids with an MOI of 1, we harvested cell-free culture media at the indicated hours postinfection, followed by RNA extraction using the MiniBEST Viral RNA/DNA Extraction Kit (TaKaRa) and detection of viral loads (viral gene copy number of RdRp gene) by one-step RT-qPCR assay, and viral titration by TCID50 assay as described previously (46, 47). All experiments with live viruses were conducted in biosafety level 3 laboratories after approval by the Faculty of Medicine, The University of Hong Kong.

### Virus competition assay.

To compare the replicative fitness of different SARS-CoV-2 strains, we infected the nasal organoid monolayers with a 1:1 mixture of WT and the Omicron variant, or a 1:1 mixture of Delta and Omicron variants at a total MOI of 0.1, or the plaque-purified each strain of the virus at an MOI of 0.1. At the indicated hours postinfection, we harvested cell-free culture media, followed by RNA extraction using the QIAamp Viral RNA minikit (Qiagen) and viral gene detection using a one-step probe-based RT-qPCR assay with a primer pair TGGACCTTGAAGGAAAACAGGG and TGGTTCTAAAGCCGAAAAACCC as described elsewhere (48, 49). The specific probe for WT and Delta variant is CTATTAATTTAGTGCGTGATCT-HEX; the specific probe for the Omicron variant is TTATAGTGCGTGAGCCAGAAGA-FAM. A LightCycler 96 system was used to simultaneously detect the FAM and HEX dye in each sample using the endpoint genotyping protocol template. The endpoint fluorescence (EPF) value of each dye in each sample was analyzed using the endpoint genotyping method with the LC96 software. EPF values of the samples from organoids infected with the purified virus (WT-only, Delta-only, or Omicron-only) were used for normalization. The percentage of each variant is calculated as the FAM/HEX value divided by the sum of FAM and HEX for each sample.

### Immunofluorescence staining and flow cytometry.

The cellular composition of the nasal organoids was characterized using specific antibodies (Table S3 in the supplemental material) to label ciliated (ACCTUB), goblet (MUC5AC), club (CC10) and basal cells (P63, CK5), followed by secondary antibodies. Cellular proteins including ACE2, TMPRSS2, OCLN, and ZO-1 in the organoids were labeled with corresponding antibodies. To identify virus-infected cells, we stained the virus-inoculated or mock-infected organoids using an in-house-made antibody against SARS-CoV-2 nucleocapsid protein (NP) raised in a guinea pig (46, 47), after fixation with 4% PFA for 1 h at room temperature, permeabilization with 0.5% Triton X-100 for 10 min and blocking with protein block (Dako) for 1 h. To define cellular tropism and to assess cilia abundance, we co-stained the infected organoids with the α-NP and α-ACCTUB. Z-stack was performed to scan nasal organoid monolayers, followed by orthogonal projection to generate the cross-sectional images. To examine cellular junctions, we co-stained the infected organoids with the α-NP and α-OCLN or α-ZO-1. Nuclei and actin filaments were counterstained with DAPI (Thermo Fisher Scientific) and Phalloidin-647 (Sigma-Aldrich), respectively. We whole-mounted the organoids on a glass slide with ProLong Glass Antifade Mountant (Invitrogen) after staining. Confocal images were acquired using a Carl Zeiss LSM 800 confocal microscope. For image quantification of cilia abundance, the area covered with cilia was manually defined and measured using the ImageJ software. Five randomly selected frames were analyzed for each group. For image quantification of cellular junction proteins, the geometric mean fluorescent intensity of each channel was measured using the Histo function of the ZEN blue software. Logarithmic binning was used; the lower and upper threshold were set to 1 and 65534, respectively.

10.1128/mbio.01944-22.7 TABLE S3 Antibody list. Download Table S3, XLSX file, 0.01 MB. Copyright © 2022 Chiu et al. 2022 Chiu et al. https://creativecommons.org/licenses/by/4.0/ This content is distributed under the terms of the Creative Commons Attribution 4.0 International license.

For flow cytometry analysis, organoids were treated with 10 mM EDTA (Invitrogen) for 30–60 min at 37°C and dissociated into single cells, followed by fixation with 4% PFA for 15 min at room temperature, permeabilization with 0.1% Triton X-100 for 5 min at 4°C, and then immunostaining using the specific antibodies (Table S3 in the supplemental material) and the corresponding isotype controls for gating. To determine infection rate, after an MOI of 1 inoculation, organoids were dissociated into single cells, fixed with 4% PFA, followed by immunostaining using an α-double-stranded RNA antibody (dsRNA, 10010500, Scicons); mock-infected organoids were used for gating. A BD FACSCantoII Analyzer or LSR Fortessa was used for analysis. FlowJo software was used for data processing.

### Scanning electron microscopy.

After infection of the indicated viruses with an MOI of 5 or mock infection, nasal organoid monolayers were mounted and coated after sequential fixation with 2.5% glutaraldehyde and 1% osmium. Sample processing was performed by Electron Microscope Unit of the University of Hong Kong. A LEO 1530 FEG Scanning Electron Microscope was used for image acquisition.

### Statistical analysis.

Statistical analysis was conducted using GraphPad Prism 9.0. Student's *t* test or ANOVA test were used to determine statistical significance as specified in the figure legends. Data in the figures represent technical replicates, i.e., the mean and s.d. of a representative experiment. The number of technical replicates is indicated in figure legends. The experiments were independently performed two to four times in multiple organoid lines, as indicated in the figure legends (*, *P* ≤ 0.05; **, *P* ≤ 0.01; ***, *P* ≤ 0.001).

### Data availability.

This study includes no data deposited in external repositories.

10.1128/mbio.01944-22.3 VIDEO S1 Beating cilia in 3D differentiated nasal organoids (magnification 400x). Download Movie S1, AVI file, 2.0 MB. Copyright © 2022 Chiu et al. 2022 Chiu et al. https://creativecommons.org/licenses/by/4.0/ This content is distributed under the terms of the Creative Commons Attribution 4.0 International license.

10.1128/mbio.01944-22.4 VIDEO S2 Beating cilia in differentiated nasal organoid monolayers (magnification 400x). Download Movie S2, AVI file, 1.8 MB. Copyright © 2022 Chiu et al. 2022 Chiu et al. https://creativecommons.org/licenses/by/4.0/ This content is distributed under the terms of the Creative Commons Attribution 4.0 International license.
