# Supplementary figures and images for: Human Nasal Organoids Model SARS-CoV-2 Upper Respiratory Infection and Recapitulate the Differential Infectivity of Emerging Variants
Source: mBio. 2022 Aug 8;13(4):e01944-22. doi: 10.1128/mbio.01944-22 (PMC9426414; doi:10.1128/mbio.01944-22)

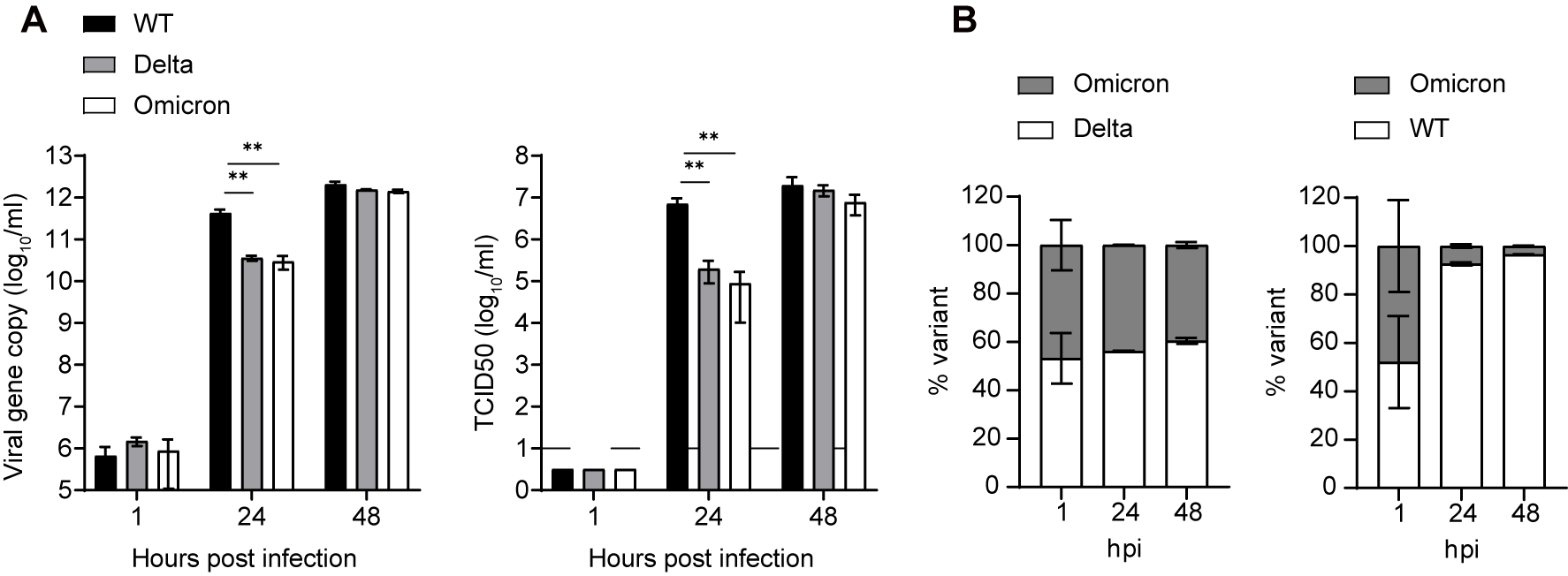

Supplement: FIG S1 [file mbio.01944-22-s0002.tif]

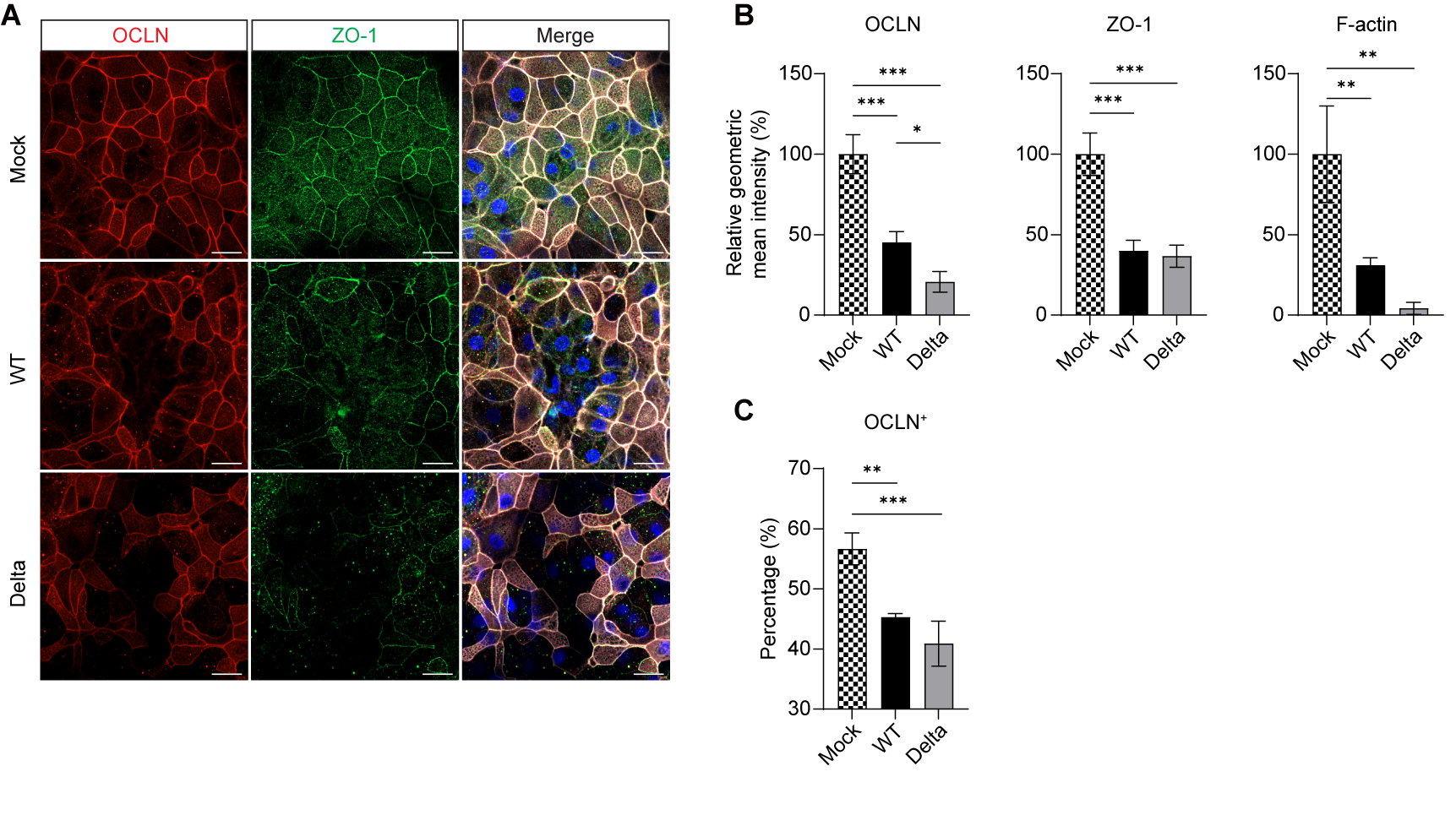

Supplement: FIG S2 [file mbio.01944-22-s0003.tif]
