# Supplementary material for: Human Nasal Organoids Model SARS-CoV-2 Upper Respiratory Infection and Recapitulate the Differential Infectivity of Emerging Variants
Source: mBio. 2022 Aug 8;13(4):e01944-22. doi: 10.1128/mbio.01944-22 (PMC9426414; doi:10.1128/mbio.01944-22)
Supplement: TEXT S1 [file mbio.01944-22-s0001.docx]

**SUPPLEMENTAL MATERIAL**

**Supplemental figures**

Figure S1.

(A) At the indicated hours post-inoculation with wildtype (WT), Delta, or Omicron variant (0.01 MOI), culture media were harvested from the Vero E6/TMPRSS2 cells and applied to viral load detection and viral titration. Data show the mean and s.d. of a representative experiment, n = 3. Multiple unpaired t-test with multiple comparisons using Holm-Sidak method. The experiment was independently performed in two organoid lines.

(B) At the indicated hours post-inoculation with a 1:1 mixture of Omicron and Delta (left), or a mixture of Omicron and WT viruses (right) (total 0.01 MOI), culture media were harvested from the Vero E6/TMPRSS2 cells and applied to viral load detection using variant-specific primers and probes. Data represent the mean and s.d. of a representative experiment, n = 3. The experiment was independently performed in two organoid lines.

Figure S2.

(A) At 24 hours post-inoculation (1 MOI), mock-, WT-, or Delta-infected airway organoid monolayer (AwO-mono) were co-stained with α-OCLN (red) and α-ZO-1(green). Nuclei and actin filaments were counterstained with DAPI (blue) and Phalloidin-647 (white). Scale bar, 20 µm.

(B) Image quantification of the immunofluorescence labeled mock-, WT-, or Delta-infected AwO-mono. Geometric mean intensity relative to mock of OCLN (left), ZO-1 (middle), and F-actin (right) were shown. Data represent the mean and s.d. of a representative experiment, n = 3. Ordinary one-way ANOVA with Tukey's multiple comparison test.

(C) At 24 hours post-inoculation (1 MOI), mock-, WT-, or Delta-infected AwO-mono were dissociated and applied to flow cytometry to detect OCLN+ cells. Data represent the mean and s.d. of a representative experiment, n = 3. Ordinary one-way ANOVA with Tukey's multiple comparison test. The experiment was independently performed in two organoid lines.

**Supplemental videos**

Video S1. Beating cilia in 3D differentiated nasal organoids (magnification 400x).

Video S2. Beating cilia in differentiated nasal organoid monolayers (magnification 400x).

**Supplemental tables**

Table S1. Composition of the expansion medium

Table S2. qPCR primer list

Table S3. Antibody list
